# Recognising and defining a new crown clade within Stromboidea Rafinesque, 1815 (Mollusca, Gastropoda)

**DOI:** 10.3897/zookeys.867.34381

**Published:** 2019-07-29

**Authors:** Stephen J. Maxwell, Aart M. Dekkers, Tasmin L. Rymer, Bradley C. Congdon

**Affiliations:** 1 College of Science and Engineering, James Cook University, Cairns Qld 4870, Australia James Cook University Cairns Australia; 2 Oasestraat 79, 1448 NR Purmerend, The Netherlands James Cook University Cairns Australia; 3 Centre for Tropical Environmental and Sustainability Sciences, James Cook University, Cairns Qld 4870, Australia James Cook University Cairns Australia

**Keywords:** Aporrhaidae, Rostellariidae, Seraphsidae, Strombidae, Struthiolariidae

## Abstract

This paper defines a new crown clade Neostromboidea to separate the Strombidae, Rostellariidae, and Seraphsidae from their sister families Struthiolariidae and Aporrhaidae. There is significant value to understanding evolutionary processes within Stromboidea to recognise the universal similarity in the position of the eye on the end of peduncles and a diminished cephalic tentacle that arises from the middle to the end on that peduncle. This is in contrast to other members of the Stromboidea where the eye is located at the base of the cephalic tentacle. These physiological differences represent two set of organisms with divergent and independent evolutionary life histories and therefore these differences need to be identifiable within the nomenclature to bring meaning to the way we name things.

## Introduction

**Figure 1.**
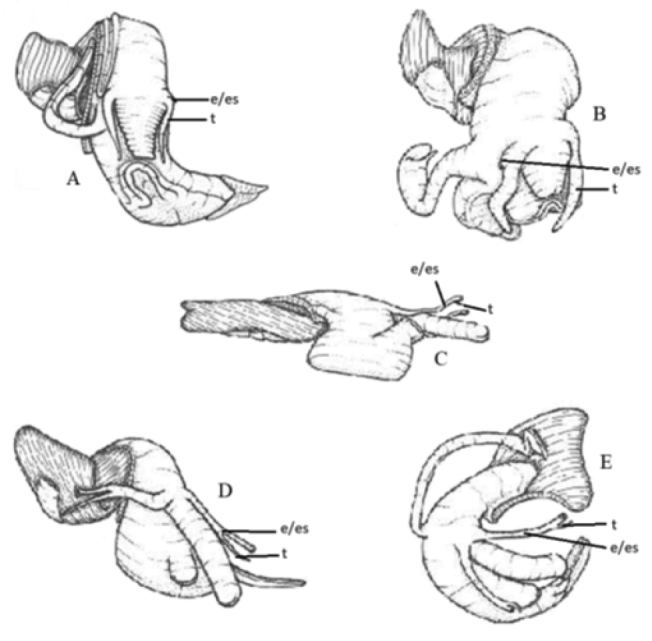
The anatomy of representatives of the five families with Stromboidea Rafinesque, 1815 indicating the eye (e) eye stalk (es) and the tentacle (t) **A**Strutholariidae Gabb, 1868 – *Tylospira scutulata* (Gmelin, 1791) (Simone 2005, fig. 254) **B**Aporrhaidae Gray, 1850 – *Aporrhais occidentalis* (Beck, 1836) (Simone 2005, fig. 297) **C**Seraphsidae Jung, 1974 – *Terebellum terebellum* (Linné, 1758) (Simone 2005, fig. 231) **D**Rostellariidae Gabb, 1868 – *Tibia insulaechorab* (Röding, 1798) (Simone 2005, fig. 249) **E**Strombidae Rafinesque, 1815 – *Strombus gallus* Linné, 1758 (Simone 2005, fig. 164).

Current Stromboidea Rafinesque, 1815 systematics has suffered from the effects of taxonomic inflation that has destroyed the evolutionary contextualisation that was once found within the historical nomenclature (Abbott 1960). This paper brings back that evolutionary contextualisation with the recognition of a new clade. There is a long history of morphologically based division with the Stromboidea. Early studies classified the Mollusca Linné, 1758 in terms of gross anatomy, with the radula being the dominating feature in some classifications (Troschel 1856–1863; Mӧrch 1866; Cooke 1895; Thiele 1931), while other classifications were based on the structure and positioning of the mantle cavity and the buccal mass, or movement of the sole of the foot (MacDonald 1857; Cooke 1885, 1927). The historically recognised recent members of the Strombidae Rafinesque, 1815 (s. l.) included the now separated Rostellariidae Gabb, 1868 and Seraphsidae Jung, 1974, both of which share a universal similarity in the positioning of the eye on the end of the peduncle, and a diminished cephalic tentacle that arises from the middle to the end on the peduncle. This contrasts with other members of the Stromboidea, the outgroups Struthiolariidae Gabb, 1868 and Aporrhaidae Gray, 1850, where the eye is located at the base of the cephalic tentacle, which is not reduced (Figure 1).

This study argues for the division of the crown clade Stromboidea based on shared morphological synapomorphies between families within this clade, which indicate a level of divergent and independent evolutionary life histories. This separation is needed to assist in resolving the higher order systematics of the Stromboidea to enable a more focused approach to understanding relationships and ancestral morphological states and patterns. There is a requirement for a name-bearing reference point that brings together the historically recognised members of the Strombidae that have now been divided into three separate families, and to distinguish those families from the other Stromboidea taxa, to achieve basal resolution of the crown clade through a clear definition and diagnosis enabling the separation from sister taxa, thus enabling an evolutionary meaning to be brought to the nomenclature of the clade.

The aim of this paper is to divide the superfamily Stromboidea by morphological evidence into two distinctive evolutionary crown clades. Crown clades are clades that are defined based on living taxa. The crown clade focussed upon here contains the families Seraphsidae, Strombidae, and Rostellariidae.

## Systematic part

### Mollusca Linné, 1758

#### Caenogastropoda Cuvier, 1797

##### Sorbeoconcha Ponder & Lindberg, 1987

###### Stromboidea Rafinesque, 1815

####### Neostromboidea new clade

Taxon classificationAnimaliaSorbeoconcha

78b4c4ac-f813-5a28-b7db-9b382b6f5acd

######## Type.

The genus *Strombus* Linné, 1758.

**Definition**: The clade is nested within Stromboidea, with the characteristics outlined in the diagnosis, and contains taxa more closely related to *Strombus pugilis* Linné, 1758 (Strombidae) *Terebellum terebellum* (Linné, 1758) (Seraphsidae) and *Tibia fusus* (Linné,1758) (Rostellariidae) than Struthiolariidae Gabb, 1868 and Aporrhaidae Gray, 1850.

**Diagnosis**: The animal possesses eyes on the end of the peduncles. The cephalic tentacle is also located on the peduncle towards the distal end. The radula has a central rachidian tooth with three lateral teeth either side. The foot is laterally compressed, with a defined propodium and a metapodium. The shell form changes upon maturation with the development of an outer lip structure.

**Figure 2.**
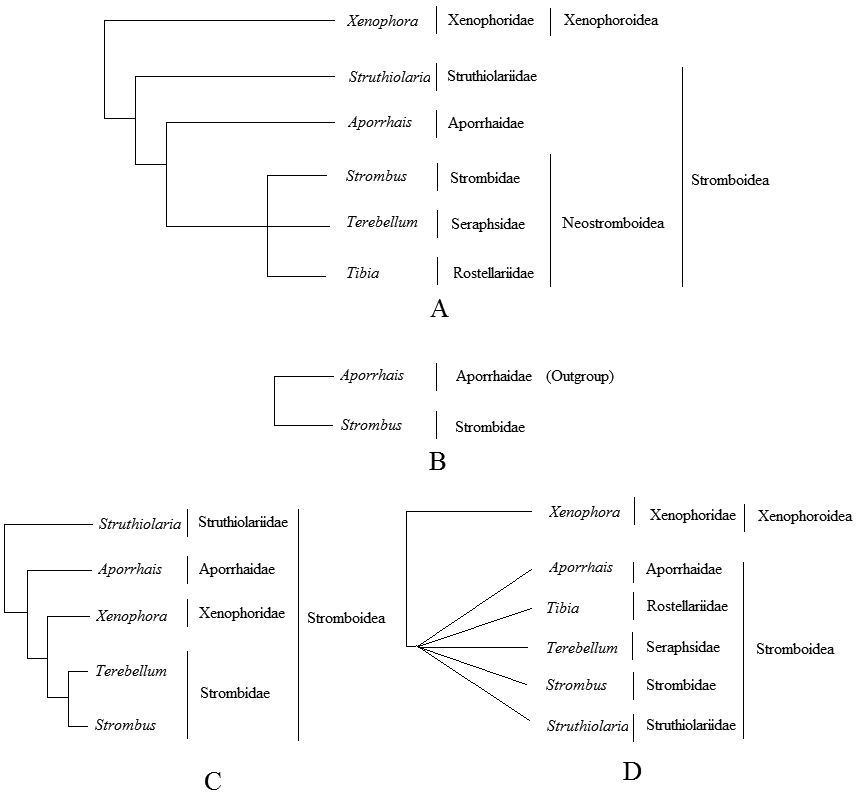
The new phylogeny of Stromboidea**A** and alternatives found from previous revisions **B** molecular analysis after Latiolais et al. (2006: 440, fig. 2) **C** anatomical analysis after Simone 2005: 261, fig. 388 **D** cladogram based on the nomenclature after MolluscaBase (2019).

**Remarks**: Neostromboidea is well supported in previous revisions and studies on the phylogeny of Stromboidea (Figure 2; Latiolais et al. 2006; Simone 2005). Simone (2005) marked this clade as “node 9” and noted that it was monophyletic within the Stromboidea. Latiolais et al. (2006) used Aporrhaidae as the outgroup in their analysis, which demonstrated a significant genetic distance between the taxa Strombidae and Aporrhaidae. Neostromboidea brings a higher level of resolution to the nomenclature by restoring the cladistic understanding and evolutionary meaning that had been lost as a consequence of taxonomic inflation (Abbott 1960; Simone 2005; MolluscaBase 2019).

## Discussion

The Neostromboidea falls within the clade Stromboidea which belongs to the highly variable invertebrate Gastropoda (Mollusca). The ancestral resolution of Neostromboidea is extremely unstable, with many conflicting views on the exact ancestors that provide a division between Neostromboidea and the two immediate outgroups Struthiolariidae and Aporrhaidae (Gabb 1869; Roy 1994; Kiel and Bandel 2002; Bandel 2007). It is postulated that *Phyllocheilus* Gabb, 1868 and *Pterodonta* d’Orbigny, 1843 form the shared common ancestor of the new clade and the Aporrhaidae and Strutholariidae based on gross morphology and the similarities with known stromboidal ancestors of Rostellariidae.

The Cretaceous clade Pugnellidae Kiel & Bandel, 1999, which is considered an ancestor of Strombidae (Wenz 1938; Sohl 1960), is somewhat fluid in its content. However, recent revisions have clarified the taxonomic position of Pugnellidae, which is now considered to be a descendant of the Aporrhaidae (Kiel and Bandel 1999). This position is based on the structure of the protoconch, the low height of the teleoconch, the lack of ornamentation typical of Strombidae, the presence of a posterior rostrum with a groove, and, importantly, the extension of the callus from the inner lip, which covers a greater portion of the teleoconch (Popenoe 1983; Kiel and Bandel 1999).

Morphologically, recent members of the Struthiolariidae and Aporrhaidae differ from Neostromboidea in having a broad rather flattened foot, as well as eyes on the base of the tentacles rather than on peduncles as with the Neostromboidea (Gardner 1875). These recent members also differ for the most part in their feeding processes, whereby the animal lies buried and extends its proboscis to ‘grasp’ at potential food items, or they are filter feeders (Purchon 1977; Savazzi 1988, 1991). However, the buried grasping feeding habit is not a significant distinguishing characteristic separating Struthiolariidae and Aporrhaidae from the Seraphsidae (Jung and Abbott 1967). Given the general instability of the aporrhaid group, it is not within the scope of this study to argue inclusiveness or provide a definition for that complex.

## Conclusions

The Neostromboidea incorporates those taxa that developed a basal sinus on the shell outer lip in conjunction with eyes placed on peduncles. The co-evolution of this shell structure and morphological trait allowed the eyestalk to protrude whilst the animal remained aperture face down on the substrate, protecting the soft parts from exposure. Furthermore, the movement of the cephalic tentacle towards the distal end of the eyestalk, thus protruding out from the basal sinus, enables the animal to achieve sensory awareness without any of the soft parts being exposed. There is much greater resolution within the Stromboidea with the recognition and naming of this clade, enabling researchers to focus on the evolution of either of the two divergent evolutionary trajectories of that clade’s members.

## Supplementary Material

XML Treatment for Neostromboidea
