# Corrigendum: Recognising and defining a new crown clade within Stromboidea Rafinesque, 1815 (Mollusca, Gastropoda). ZooKeys 867: 1–7. https://doi.org/10.3897/zookeys.867.34381

**DOI:** 10.3897/zookeys.870.38846

**Published:** 2019-08-07

**Authors:** Stephen J. Maxwell

**Affiliations:** 1 College of Science and Engineering, James Cook University, Cairns Qld 4870, Australia James Cook University Cairns Australia

## Abstract

The paper recongising a new crown clade within Stromboidea contains typographic errors that occurred during copy editing: &ldquo;Neostromboidea&rdquo; should read &ldquo;Neostromboidae&rdquo; throughout. Neostromboidae is an epifamily.

The paper “Recognising and defining a new crown clade within Stromboidea Rafinesque, 1815 (Mollusca, Gastropoda)” contains a typographic error that occurred during copy editing: “Neostromboidea” should read “Neostromboidae”. “-oidae” is the most correct epifamily rank declension in this case.

